# Supplementary material for: Risk factors for abdominal aortic aneurysm in general populations: A systematic review and meta-analysis
Source: PLoS One. 2025 Sep 22;20(9):e0329500. doi: 10.1371/journal.pone.0329500 (PMC12453230; doi:10.1371/journal.pone.0329500)
Supplement: S1 File — (DOCX) [file pone.0329500.s001.docx]

**Search strategy**

**Pubmed:**

("Abdominal Aortic Aneurysms" [Mesh] OR "Aneurysms, Abdominal Aortic" [Mesh] OR "Aortic Aneurysms, Abdominal" [Mesh] OR "Abdominal Aortic Aneurysm" [Title/Abstract] OR "Aneurysm, Abdominal Aortic" [Title/Abstract]) AND (("Screening" [Mesh] OR "Mass Screenings" [Mesh] OR "Screening, Mass" [Title/Abstract] OR "Screenings, Mass" [Title/Abstract] OR "Screenings" [Title/Abstract]) OR ("Factor, Risk" [Mesh] OR "Factors, Risk" [Title/Abstract] OR "Risk Factor" [Title/Abstract] OR "Population at Risk" [Title/Abstract] OR "Risk, Population at" [Title/Abstract] OR "Populations at Risk" [Title/Abstract] OR "Risk, Populations at" [Title/Abstract])) NOT "surgical repair" [Title/Abstract]

**EmBase:**

('abdominal aortic aneurysm'/exp OR 'aneurysms, abdominal aortic'/exp OR 'aortic aneurysms, abdominal'/exp OR 'abdominal aortic aneurysm':ti,ab OR 'aneurysm, abdominal aortic':ti,ab) AND ('screening'/exp OR 'mass screenings'/exp OR 'screening, mass':ti,ab OR 'screenings, mass':ti,ab OR 'screenings':ti,ab) AND ('risk factor'/exp OR 'factors, risk':ti,ab OR 'risk factor':ti,ab OR 'population at risk':ti,ab OR 'risk, population at':ti,ab OR 'populations at risk':ti,ab OR 'risk, populations at':ti,ab) NOT 'surgical repair':ti,ab

**Cochrane library:**

(ME "Abdominal Aortic Aneurysms" OR ME "Aneurysms, Abdominal Aortic" OR ME "Aortic Aneurysms, Abdominal" OR "Abdominal Aortic Aneurysm" OR "Aneurysm, Abdominal Aortic") AND (ME "Screening" OR ME "Mass Screenings" OR "Screening, Mass" OR "Screenings, Mass" OR "Screenings") AND (ME "Risk Factors" OR "Factor, Risk" OR "Factors, Risk" OR "Risk Factor" OR "Population at Risk" OR "Risk, Population at" OR "Populations at Risk" OR "Risk, Populations at")

NOT "surgical repair"
