# Supplementary material for: Risk factors for abdominal aortic aneurysm in general populations: A systematic review and meta-analysis
Source: PLoS One. 2025 Sep 22;20(9):e0329500. doi: 10.1371/journal.pone.0329500 (PMC12453230; doi:10.1371/journal.pone.0329500)
Supplement: S1 Table — (DOCX) [file pone.0329500.s005.docx]

S1 Table. Quality scores of prospective cohort studies using Newcastle-Ottawa Scale.

| Study | Selection | | | | Comparability | Outcome | | | NOS |
| --- | --- | --- | --- | --- | --- | --- | --- | --- | --- |
|  | Representativeness of the exposed cohort | Selection of the non exposed cohort | Ascertainment  of exposure | Demonstration that outcomes was not present at start of study | Comparability on the basis of the design or analysis | Assessment of outcome | Adequate follow-up duration | Adequate follow-up rate | Overall score |
| Smith 1993 [23] | 1 | 1 | 1 | 1 | 2 | 1 | 0 | 0 | 7 |
| Simoni 1995 [24] | 1 | 1 | 1 | 1 | 1 | 1 | 0 | 0 | 6 |
| Lederle 1997 [25] | 1 | 1 | 1 | 1 | 2 | 1 | 0 | 1 | 8 |
| Vazquez 1998 [26] | 0 | 1 | 1 | 1 | 2 | 1 | 0 | 0 | 6 |
| Lederle 2000 [27] | 1 | 1 | 1 | 1 | 2 | 1 | 0 | 1 | 8 |
| Singh 2001 [28] | 1 | 1 | 1 | 1 | 2 | 1 | 0 | 1 | 8 |
| Kent 2010 [29] | 1 | 1 | 1 | 1 | 1 | 1 | 0 | 1 | 7 |
| Svensjo 2011 [30] | 1 | 1 | 1 | 1 | 2 | 1 | 0 | 0 | 7 |
| Barba 2013 [31] | 0 | 1 | 1 | 1 | 2 | 1 | 0 | 0 | 6 |
| Hager 2013 [32] | 1 | 1 | 1 | 1 | 2 | 1 | 0 | 0 | 7 |
| Svensjo 2013 [33] | 1 | 1 | 1 | 1 | 2 | 1 | 0 | 0 | 7 |
| Jawien 2014 [34] | 0 | 1 | 1 | 1 | 2 | 1 | 0 | 0 | 6 |
| Chun 2014 [35] | 1 | 1 | 1 | 1 | 1 | 1 | 0 | 1 | 7 |
| Bohlin 2014 [36] | 1 | 1 | 1 | 1 | 1 | 1 | 0 | 1 | 7 |
| Golledge 2014 [37] | 1 | 1 | 1 | 1 | 2 | 1 | 0 | 0 | 7 |
| Jahangir 2015 [38] | 1 | 1 | 1 | 1 | 2 | 1 | 0 | 1 | 8 |
| Salvador-Gonzalez 2016 [39] | 0 | 1 | 1 | 1 | 2 | 1 | 0 | 0 | 6 |
| Corrado 2016 [40] | 1 | 1 | 1 | 1 | 1 | 1 | 0 | 1 | 7 |
| Kvist 2017 [41] | 1 | 1 | 1 | 1 | 1 | 1 | 0 | 1 | 7 |
| Siso-Almirall 2017 [42] | 1 | 1 | 1 | 1 | 2 | 1 | 0 | 1 | 8 |
| Stackelberg 2017 [43] | 1 | 1 | 1 | 1 | 2 | 1 | 0 | 1 | 8 |
| Han 2017 [44] | 1 | 1 | 1 | 1 | 1 | 1 | 0 | 0 | 6 |
| Li 2018 [45] | 1 | 1 | 1 | 1 | 2 | 1 | 0 | 0 | 7 |
| Kilic 2018 [46] | 1 | 1 | 1 | 1 | 1 | 1 | 0 | 0 | 6 |
| Song 2020 [47] | 1 | 1 | 1 | 1 | 1 | 1 | 0 | 0 | 6 |
| Vats 2020 [48] | 0 | 1 | 1 | 1 | 1 | 1 | 0 | 1 | 6 |
| Summers 2021 [49] | 1 | 1 | 1 | 1 | 1 | 1 | 0 | 1 | 7 |
| Wiles 2021 [50] | 0 | 1 | 1 | 1 | 1 | 1 | 0 | 1 | 6 |
| Obel 2021 [51] | 1 | 1 | 1 | 1 | 2 | 1 | 0 | 1 | 8 |
| Kim 2023 [52] | 1 | 1 | 1 | 1 | 1 | 1 | 0 | 0 | 6 |
| Lin 2023 [53] | 1 | 1 | 1 | 1 | 1 | 1 | 0 | 1 | 7 |
| Koncar 2024 [54] | 0 | 1 | 1 | 1 | 1 | 1 | 0 | 1 | 6 |
| Stacey 2024 [55] | 1 | 1 | 1 | 1 | 1 | 1 | 0 | 1 | 7 |
| Persson 2025 [56] | 1 | 1 | 1 | 1 | 2 | 1 | 0 | 1 | 8 |
