# Supplementary figures and images for: Risk factors for abdominal aortic aneurysm in general populations: A systematic review and meta-analysis
Source: PLoS One. 2025 Sep 22;20(9):e0329500. doi: 10.1371/journal.pone.0329500 (PMC12453230; doi:10.1371/journal.pone.0329500)

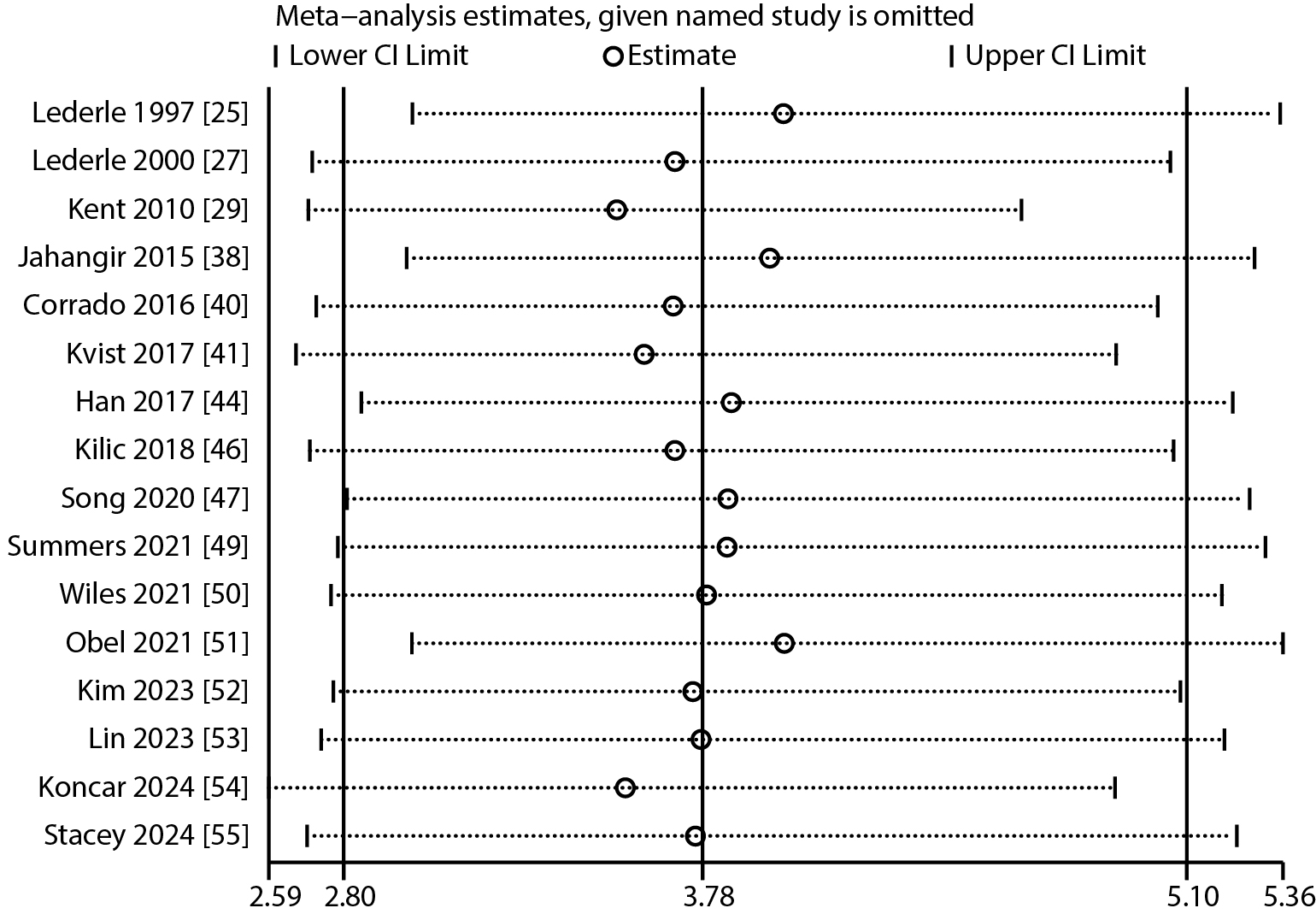

Supplement: S1 Fig — (JPG) [file pone.0329500.s006.jpg]

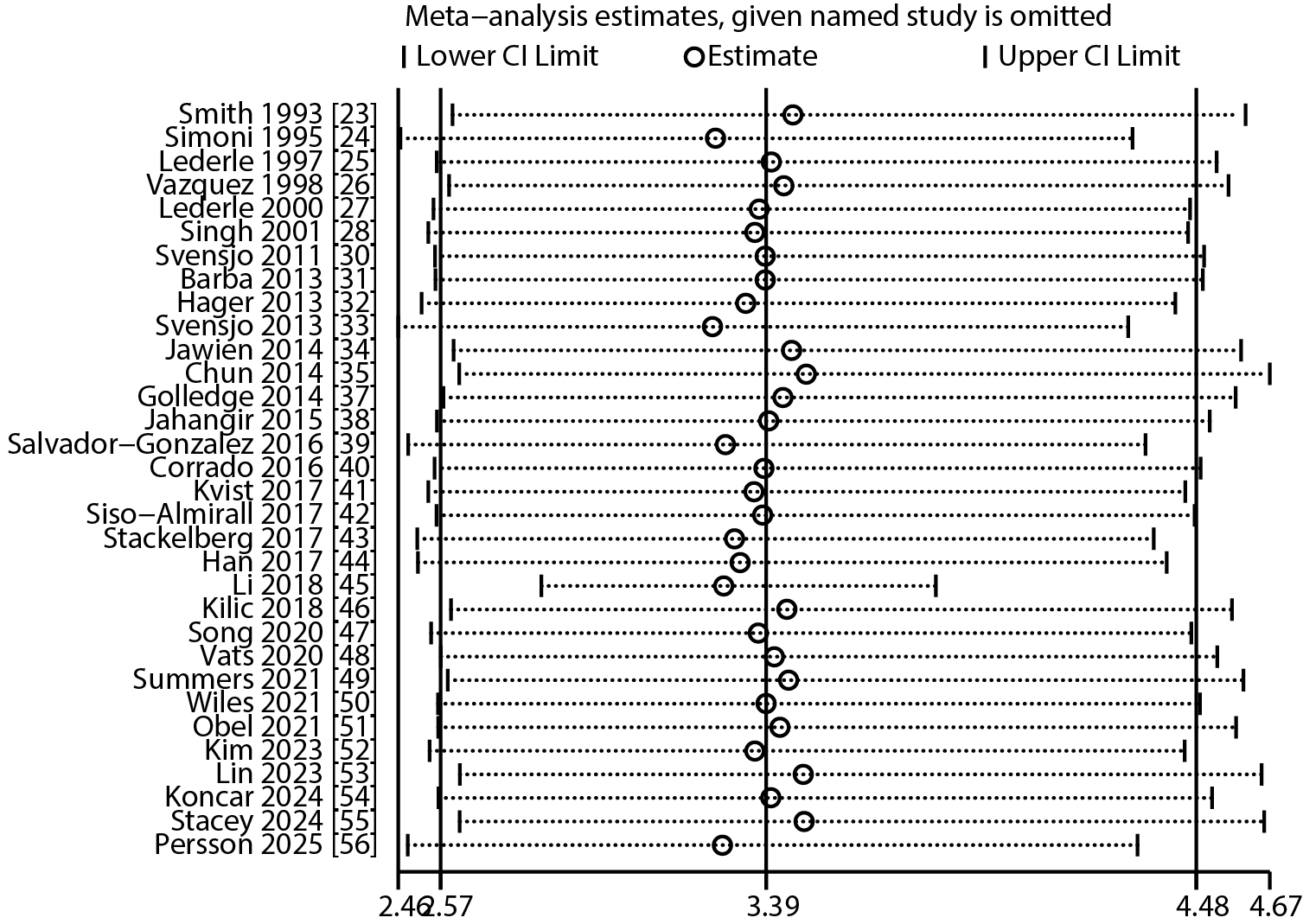

Supplement: S2 Fig — (JPG) [file pone.0329500.s007.jpg]

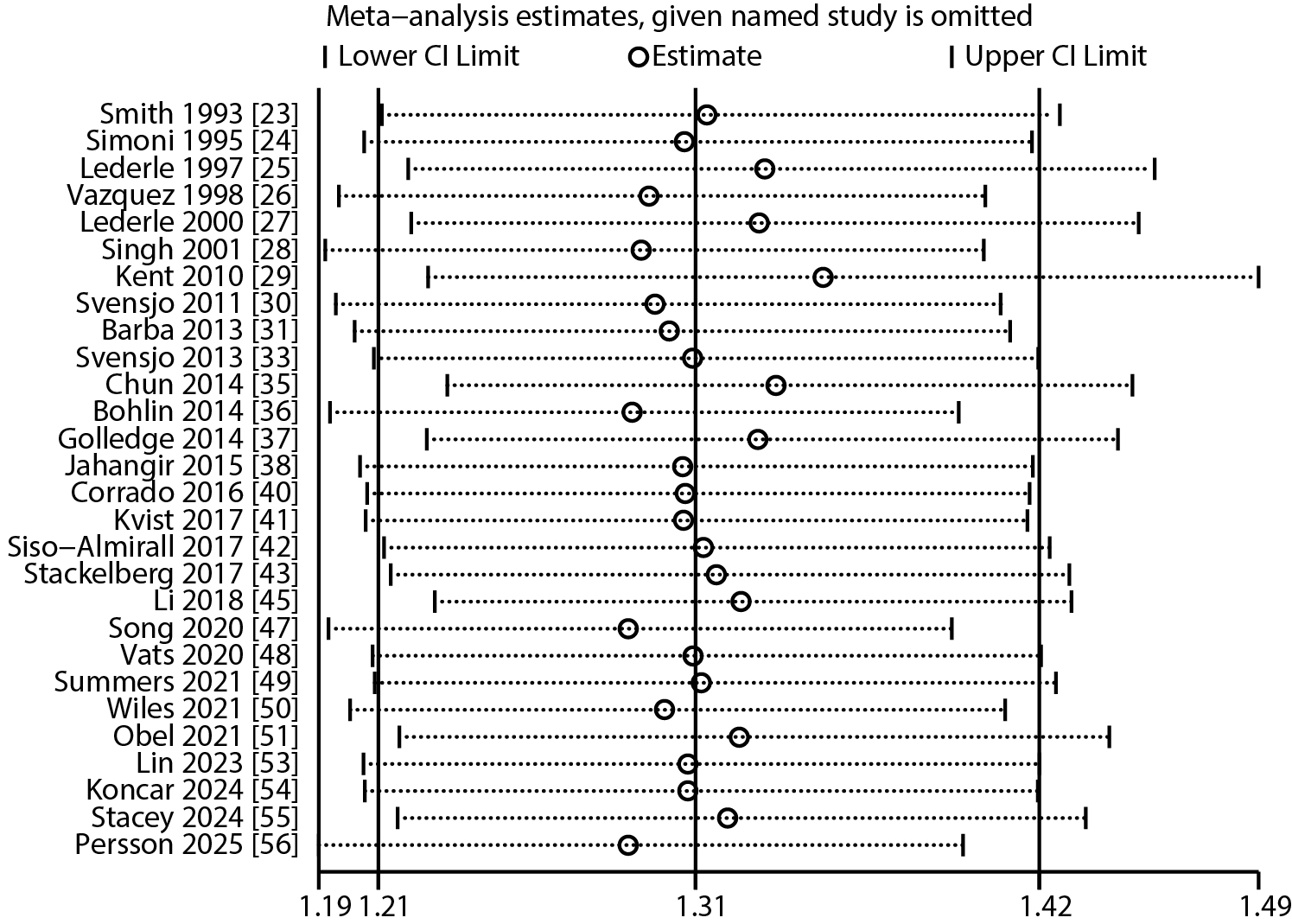

Supplement: S3 Fig — (JPG) [file pone.0329500.s008.jpg]

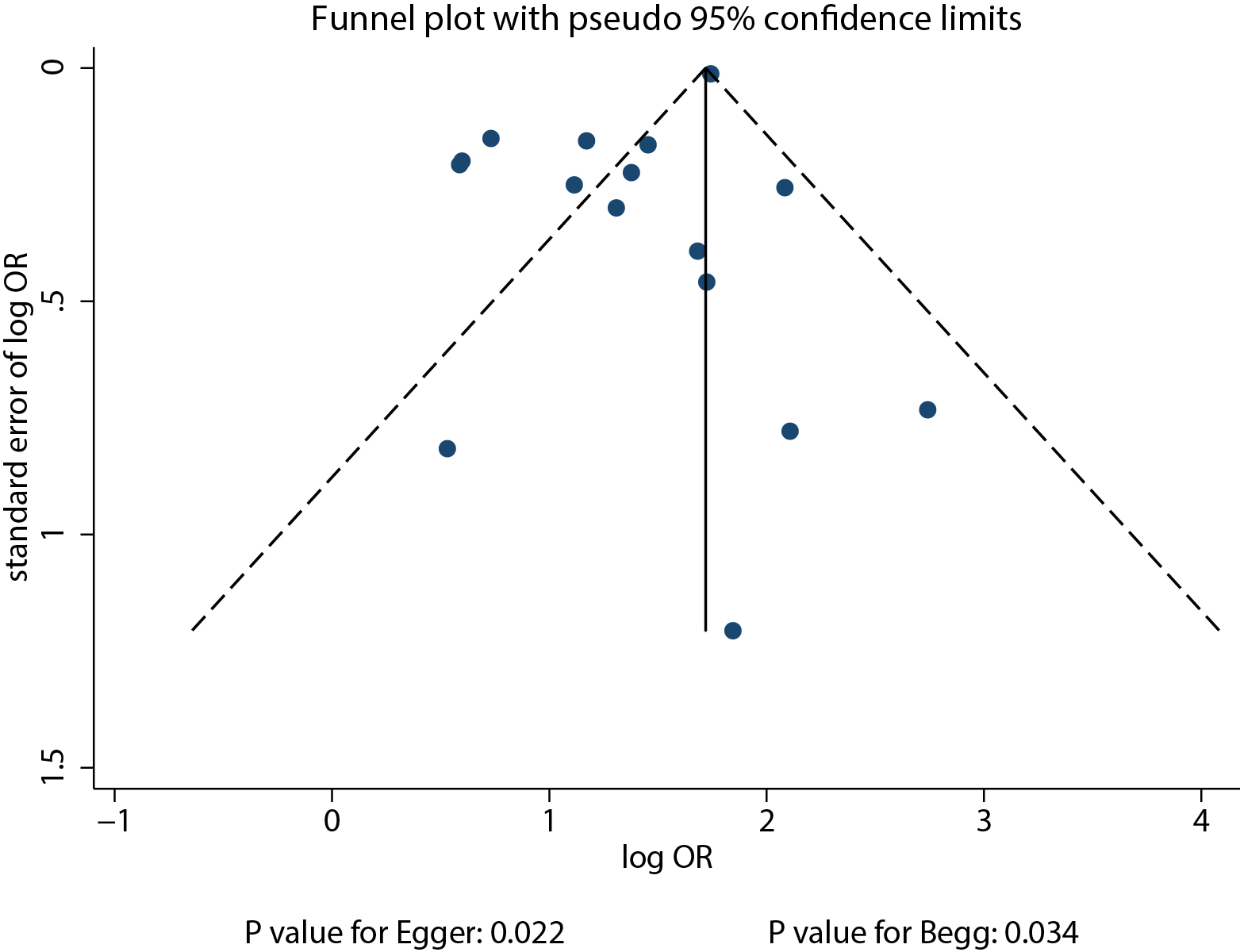

Supplement: S4 Fig — (JPG) [file pone.0329500.s009.jpg]

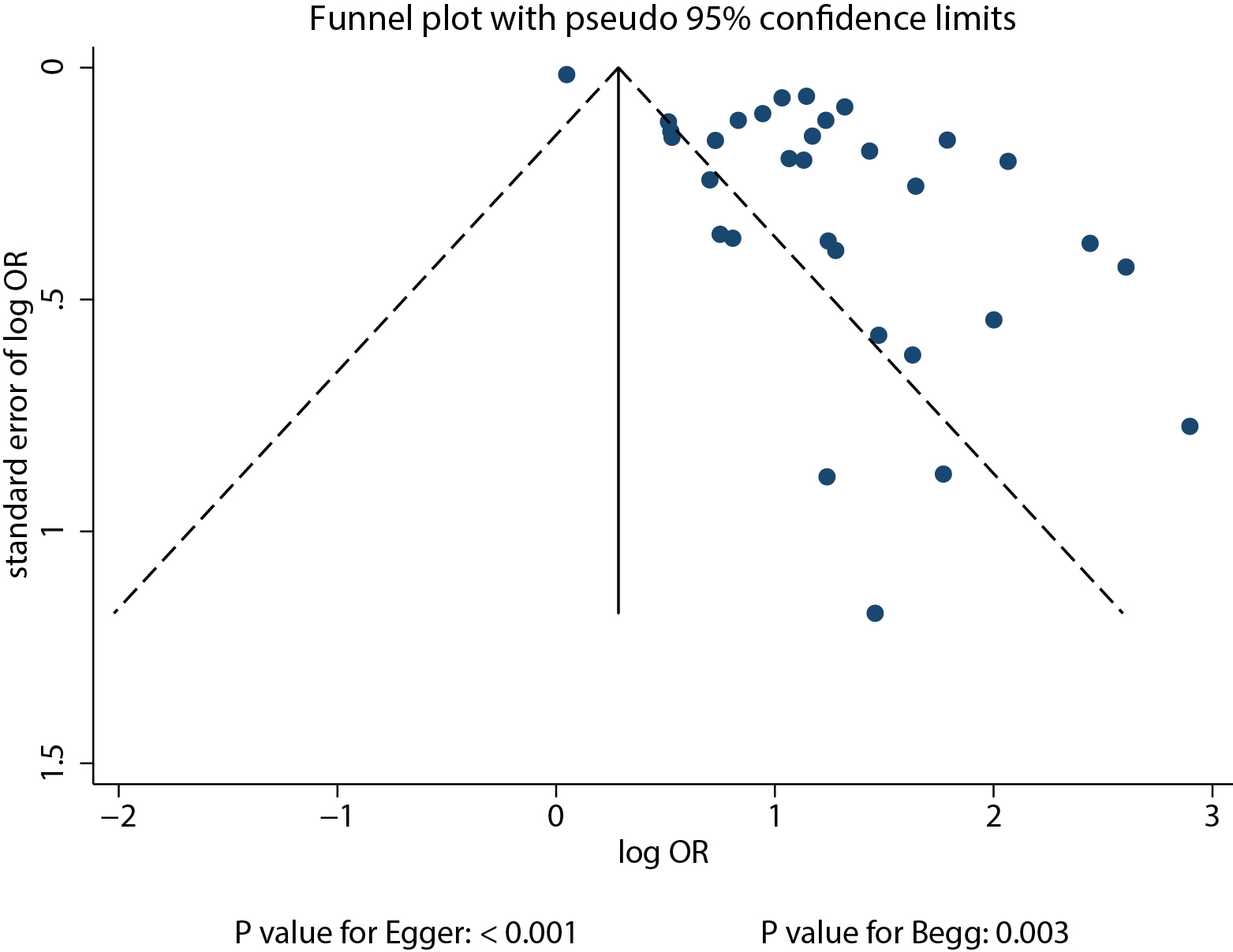

Supplement: S5 Fig — (JPG) [file pone.0329500.s010.jpg]

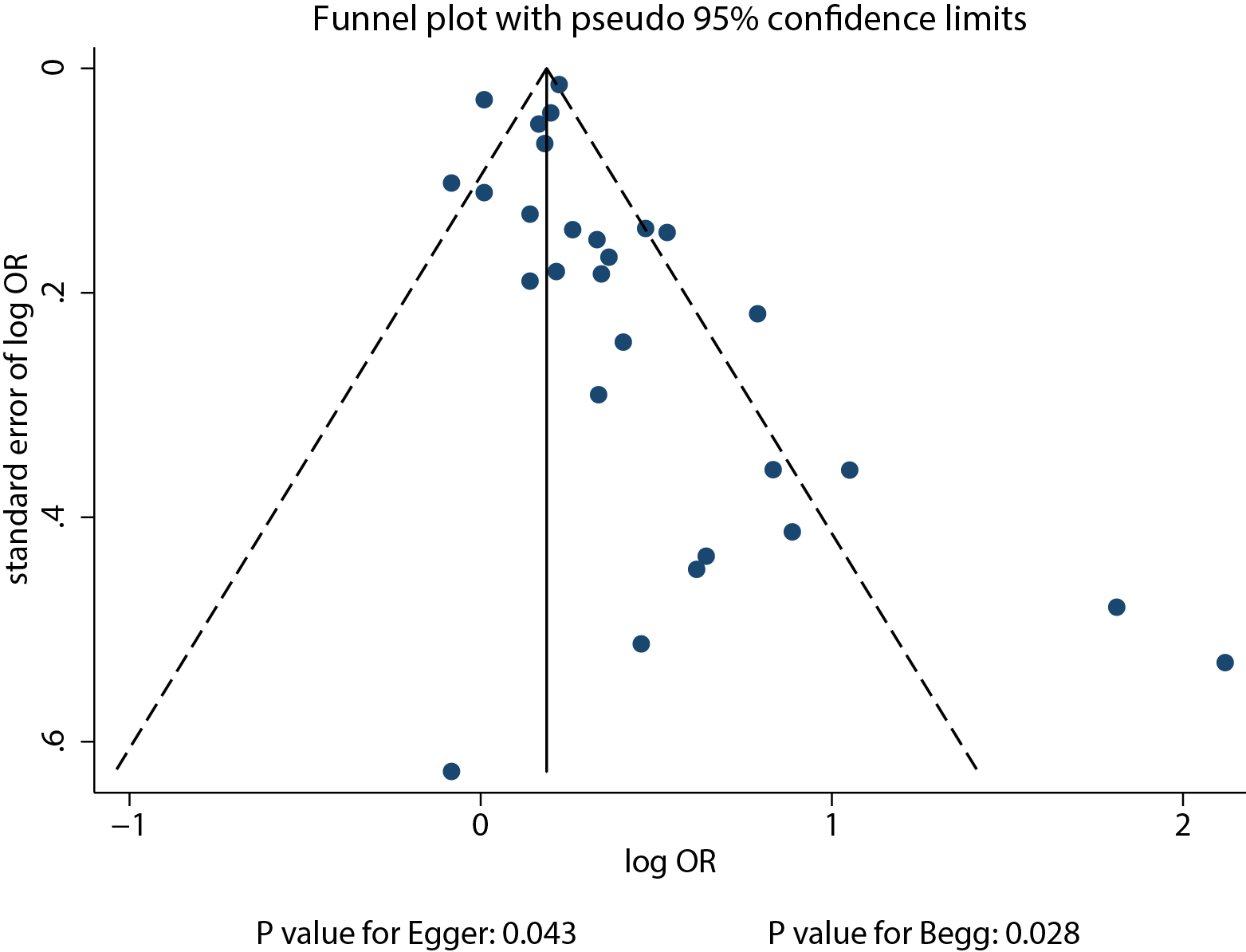

Supplement: S6 Fig — (JPG) [file pone.0329500.s011.jpg]

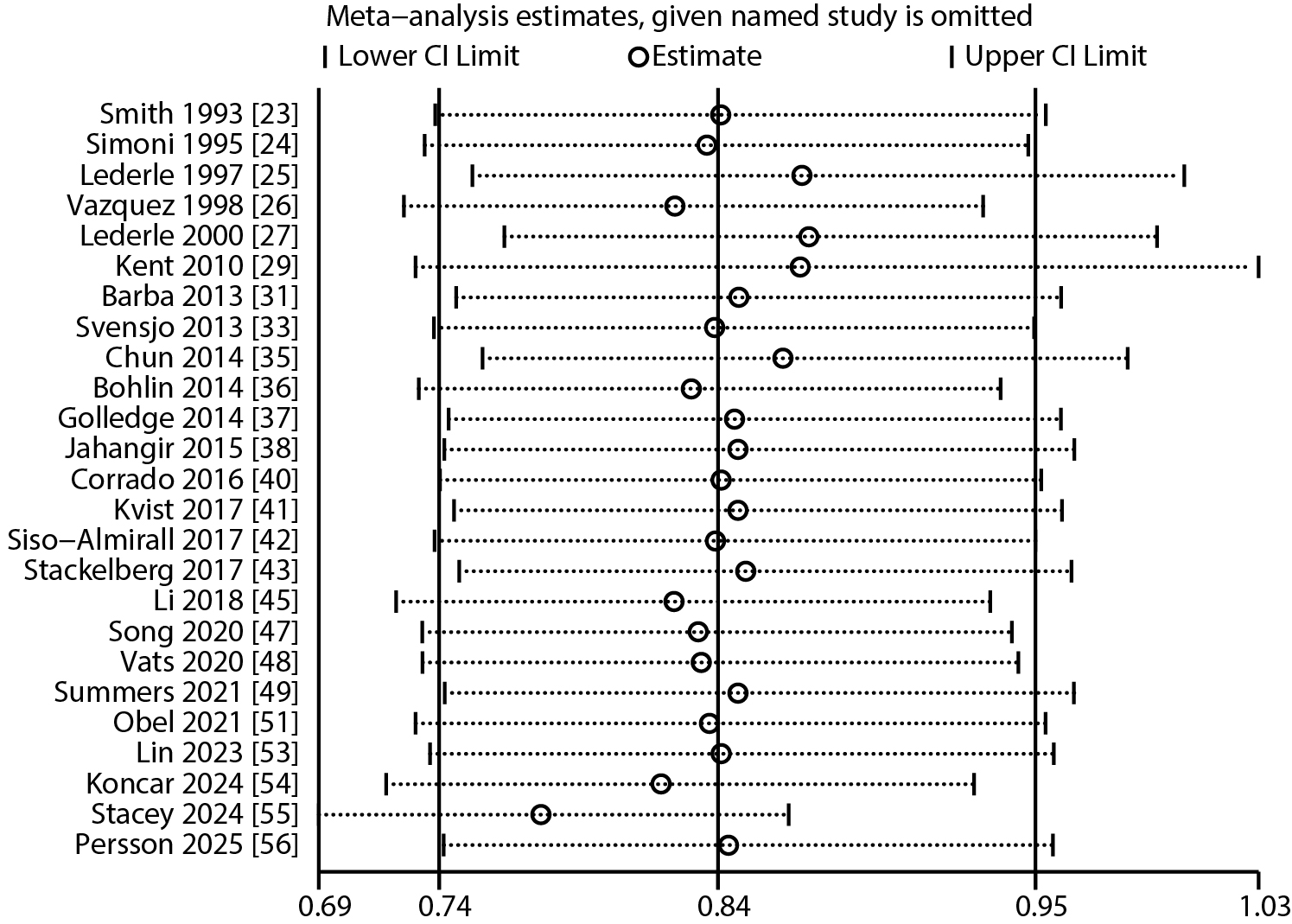

Supplement: S7 Fig — (JPG) [file pone.0329500.s012.jpg]

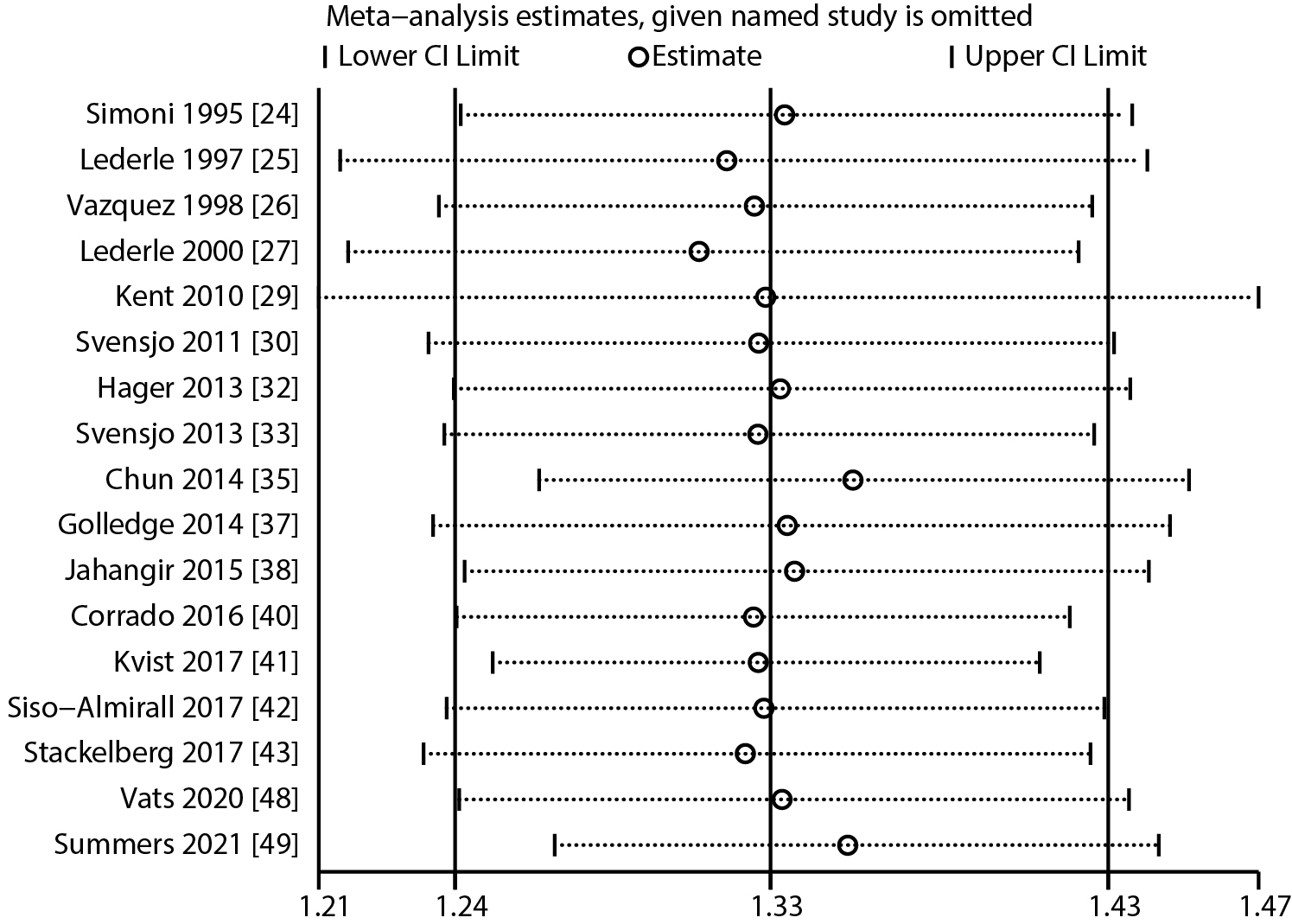

Supplement: S8 Fig — (JPG) [file pone.0329500.s013.jpg]

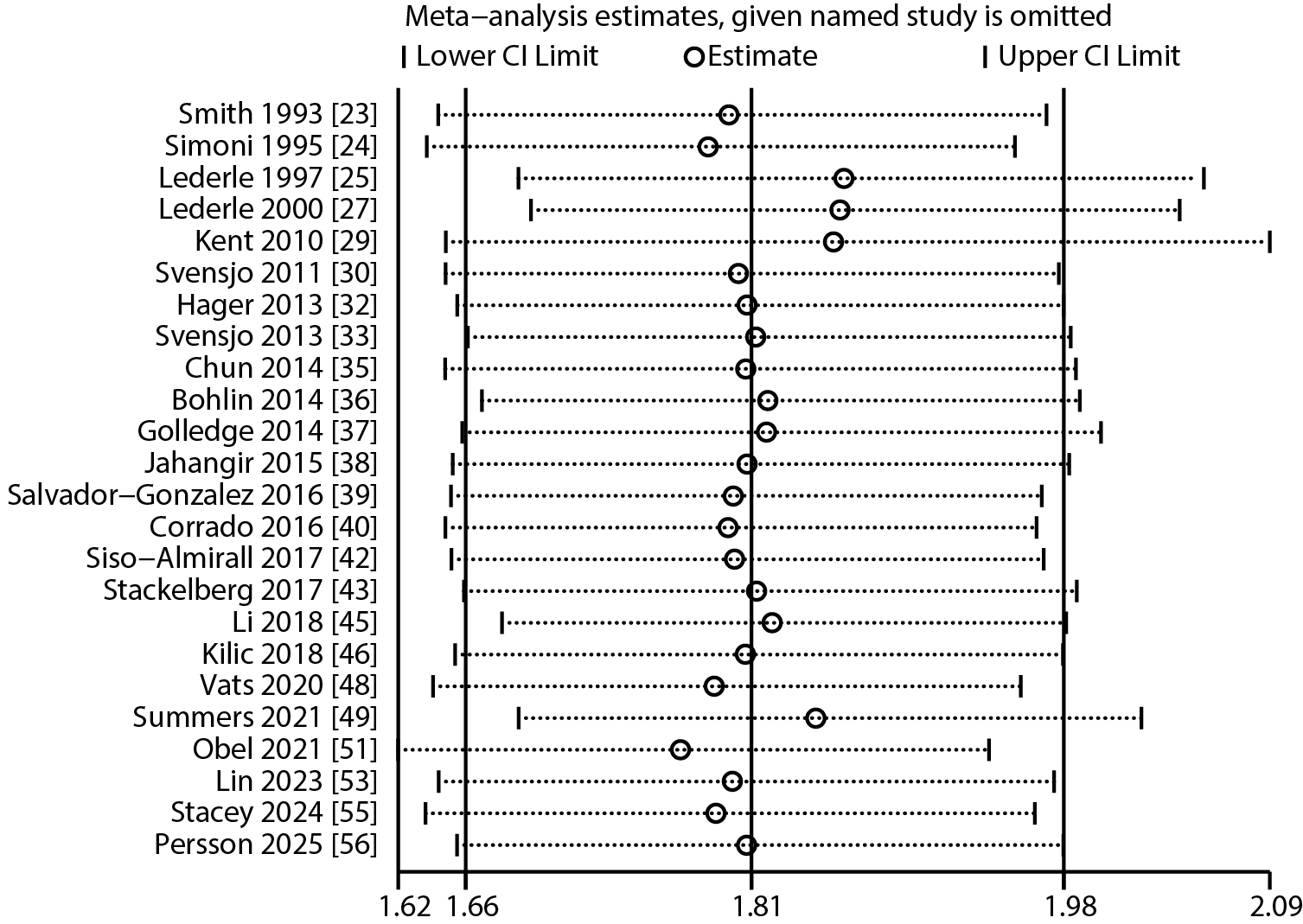

Supplement: S9 Fig — (JPG) [file pone.0329500.s014.jpg]

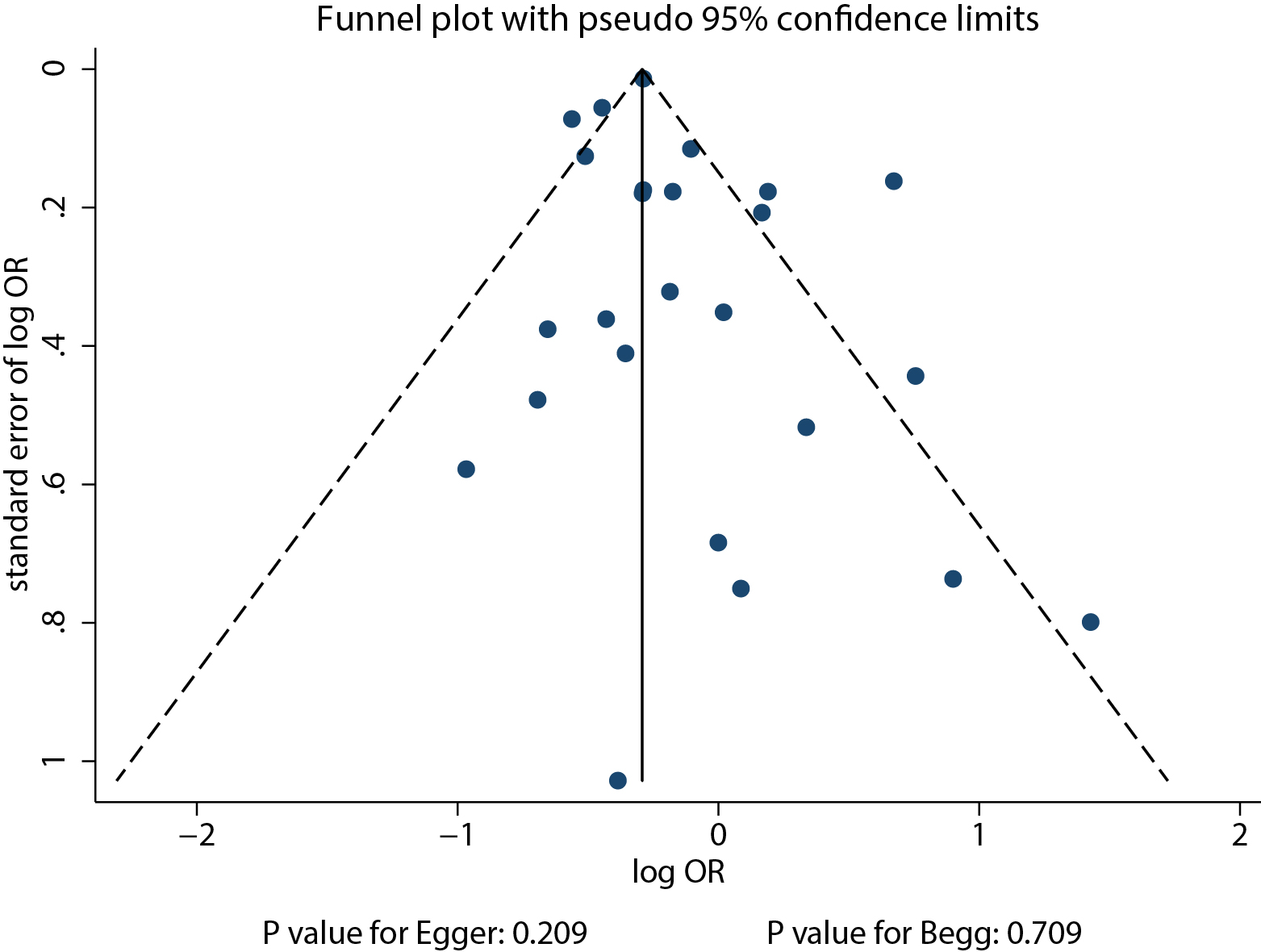

Supplement: S10 Fig — (JPG) [file pone.0329500.s015.jpg]

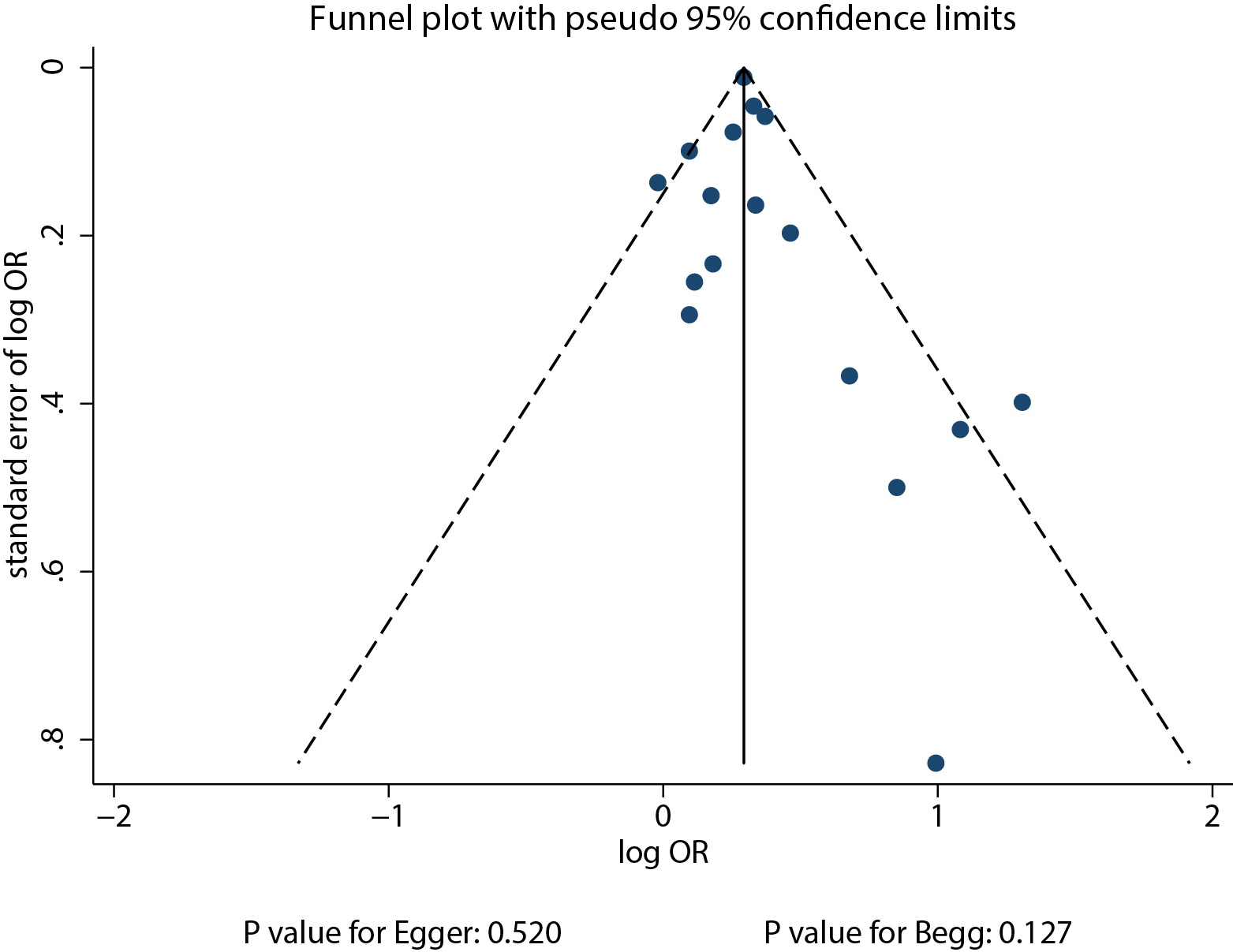

Supplement: S11 Fig — (JPG) [file pone.0329500.s016.jpg]

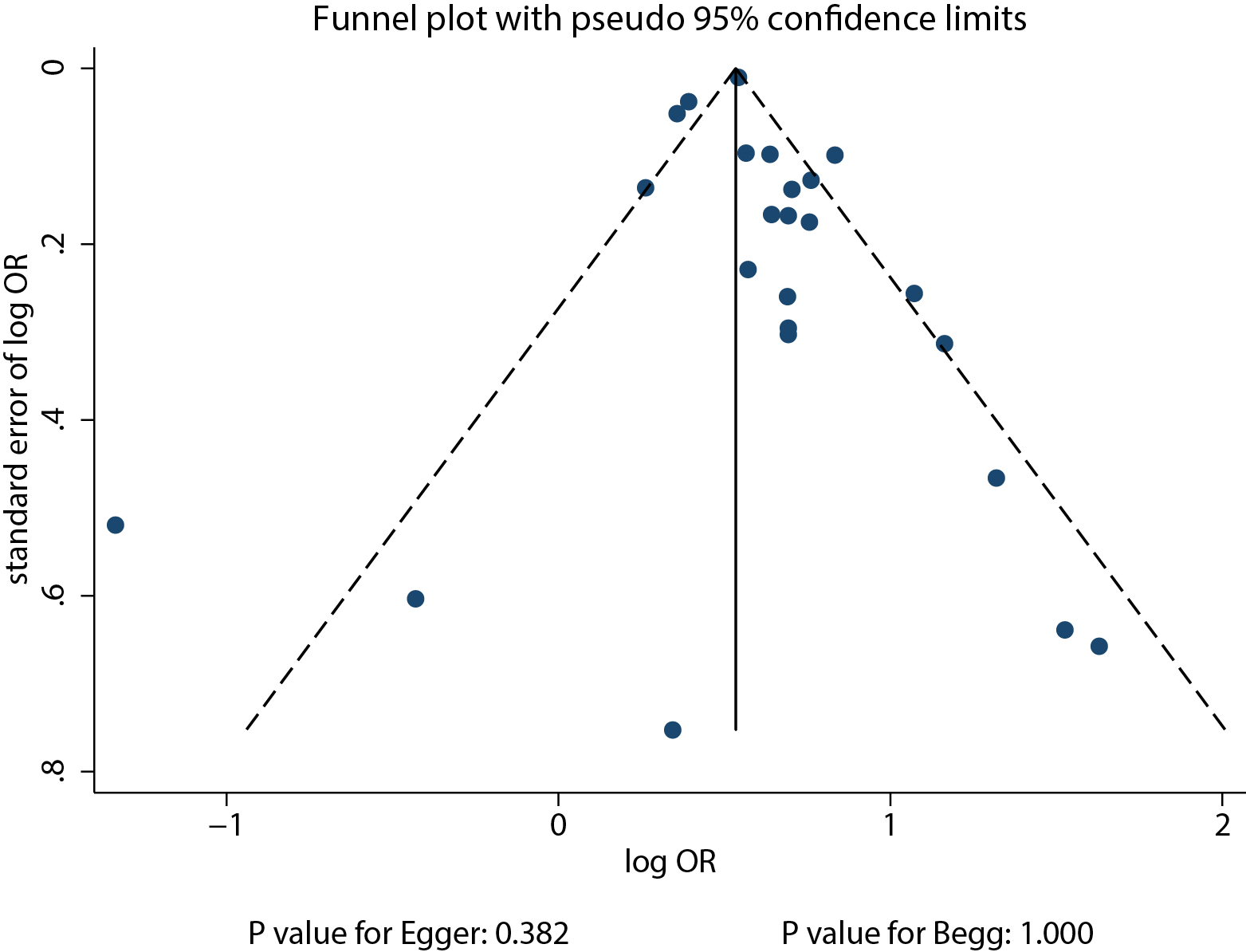

Supplement: S12 Fig — (JPG) [file pone.0329500.s017.jpg]

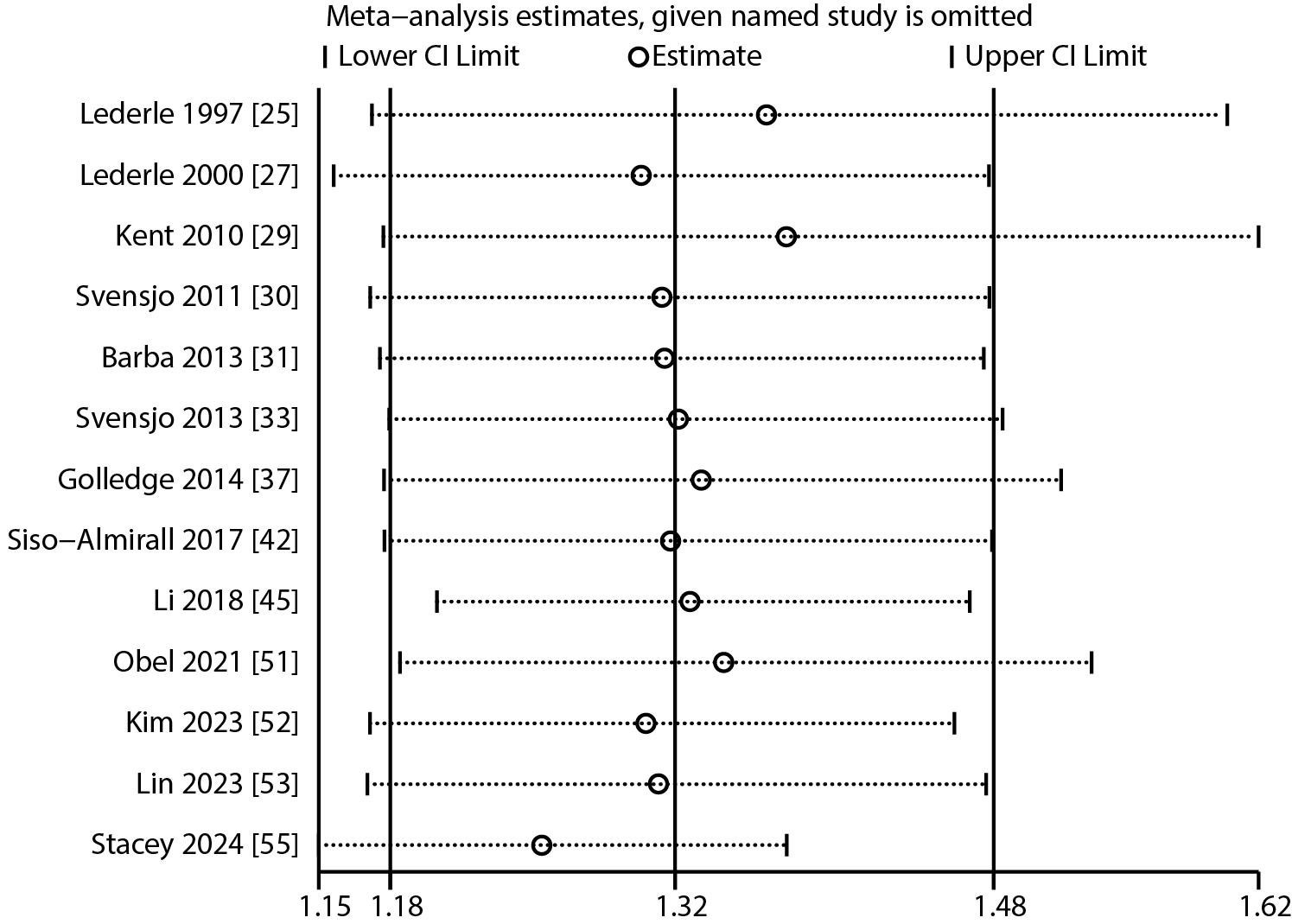

Supplement: S13 Fig — (JPG) [file pone.0329500.s018.jpg]

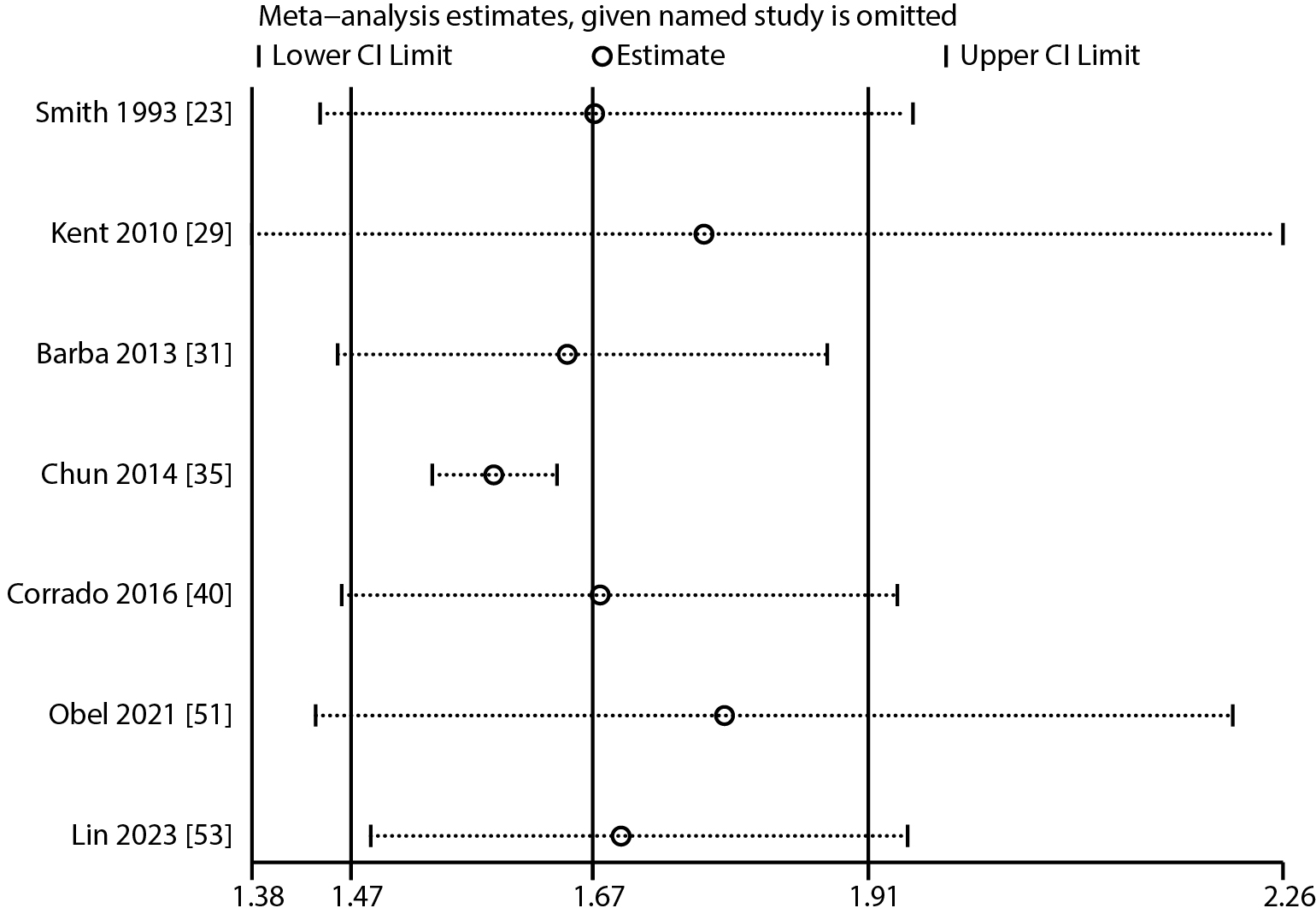

Supplement: S14 Fig — (JPG) [file pone.0329500.s019.jpg]

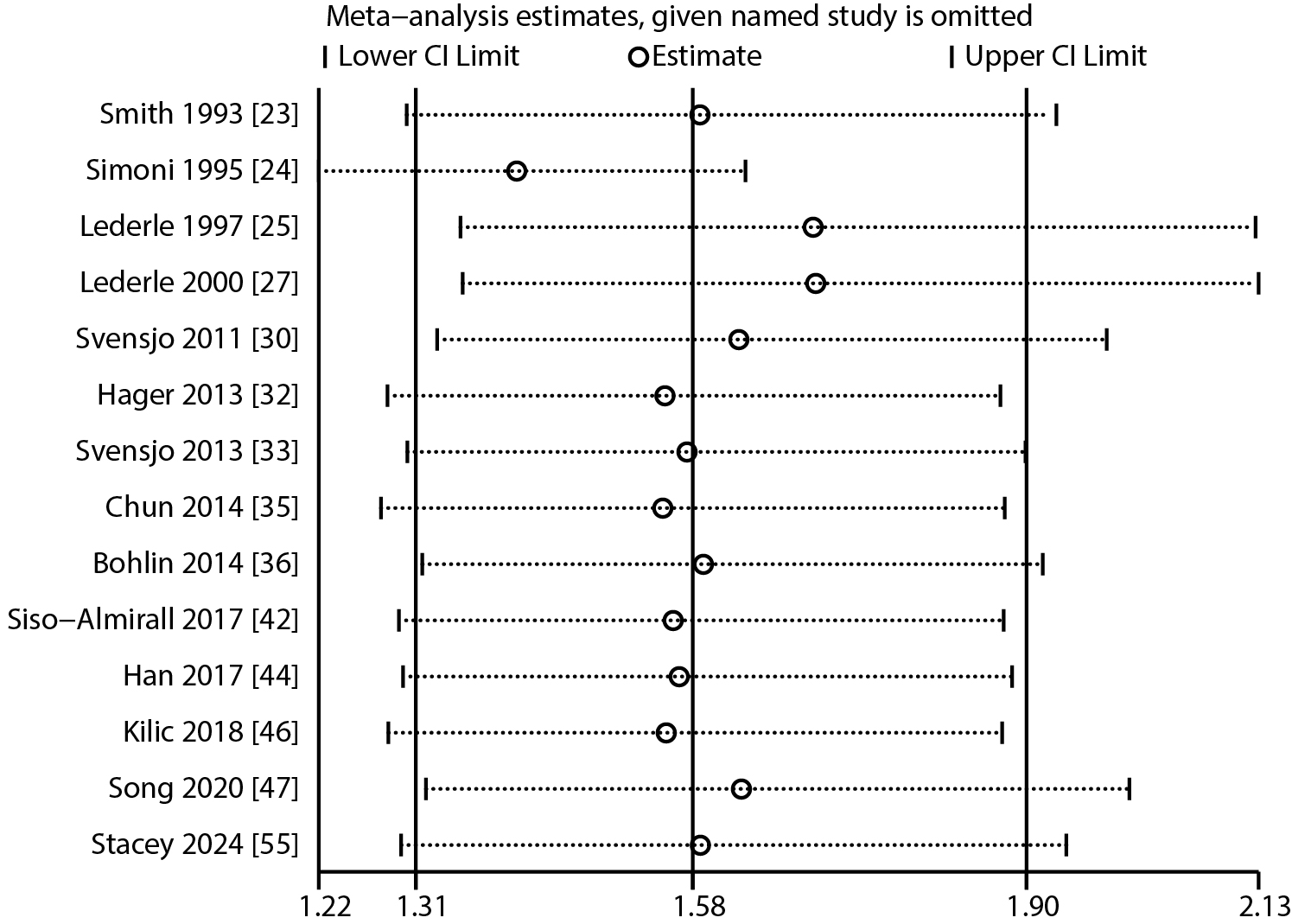

Supplement: S15 Fig — (JPG) [file pone.0329500.s020.jpg]

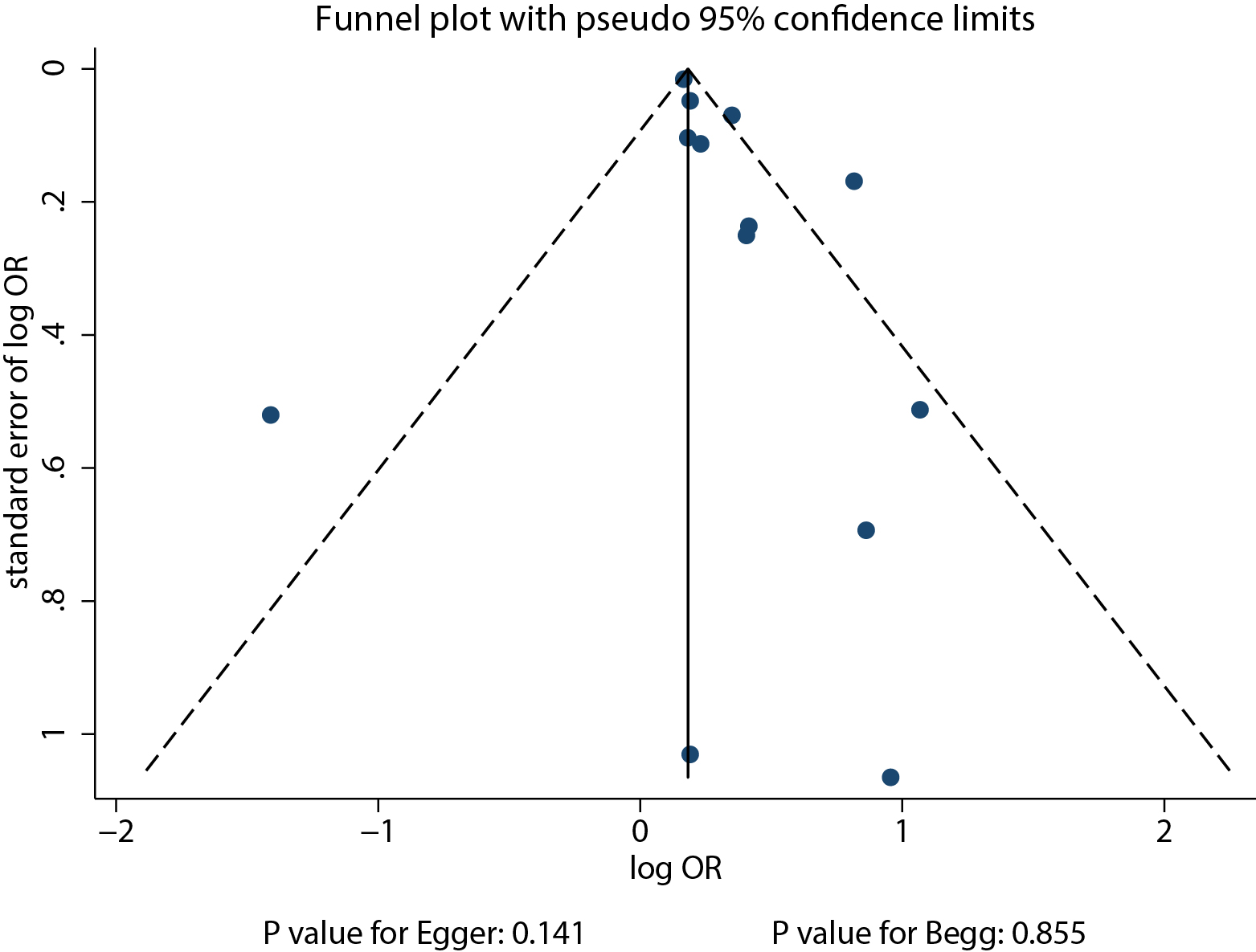

Supplement: S16 Fig — (JPG) [file pone.0329500.s021.jpg]

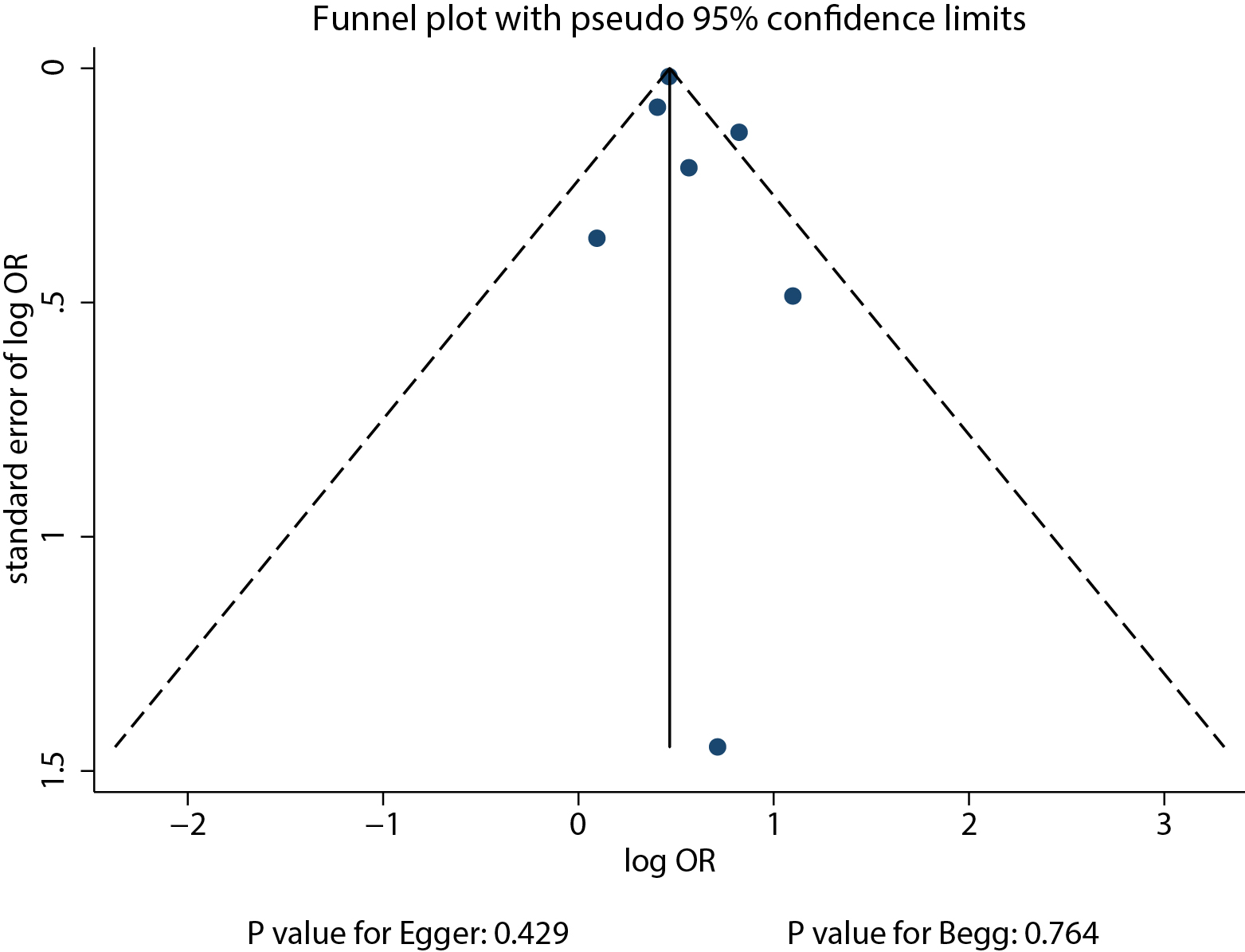

Supplement: S17 Fig — (JPG) [file pone.0329500.s022.jpg]

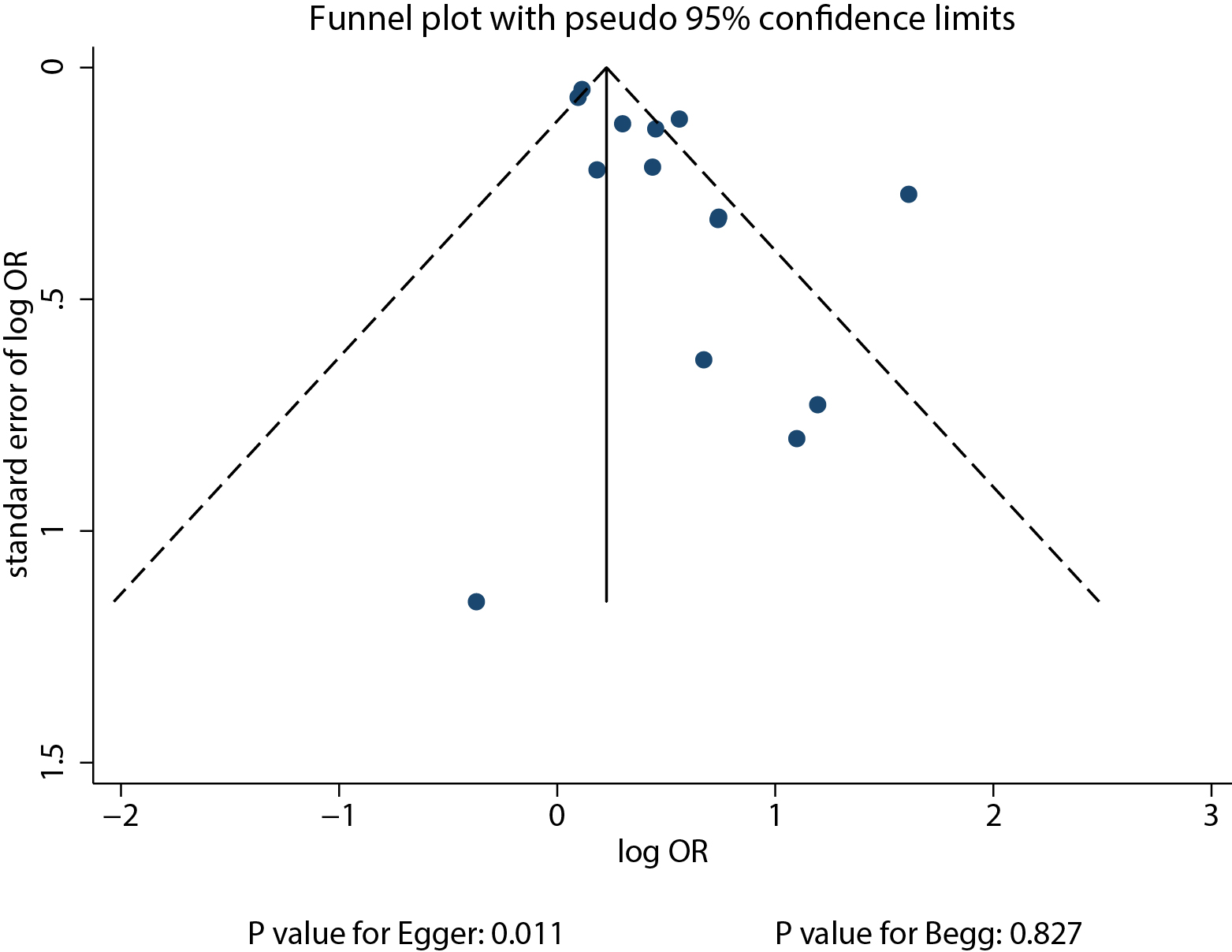

Supplement: S18 Fig — (JPG) [file pone.0329500.s023.jpg]

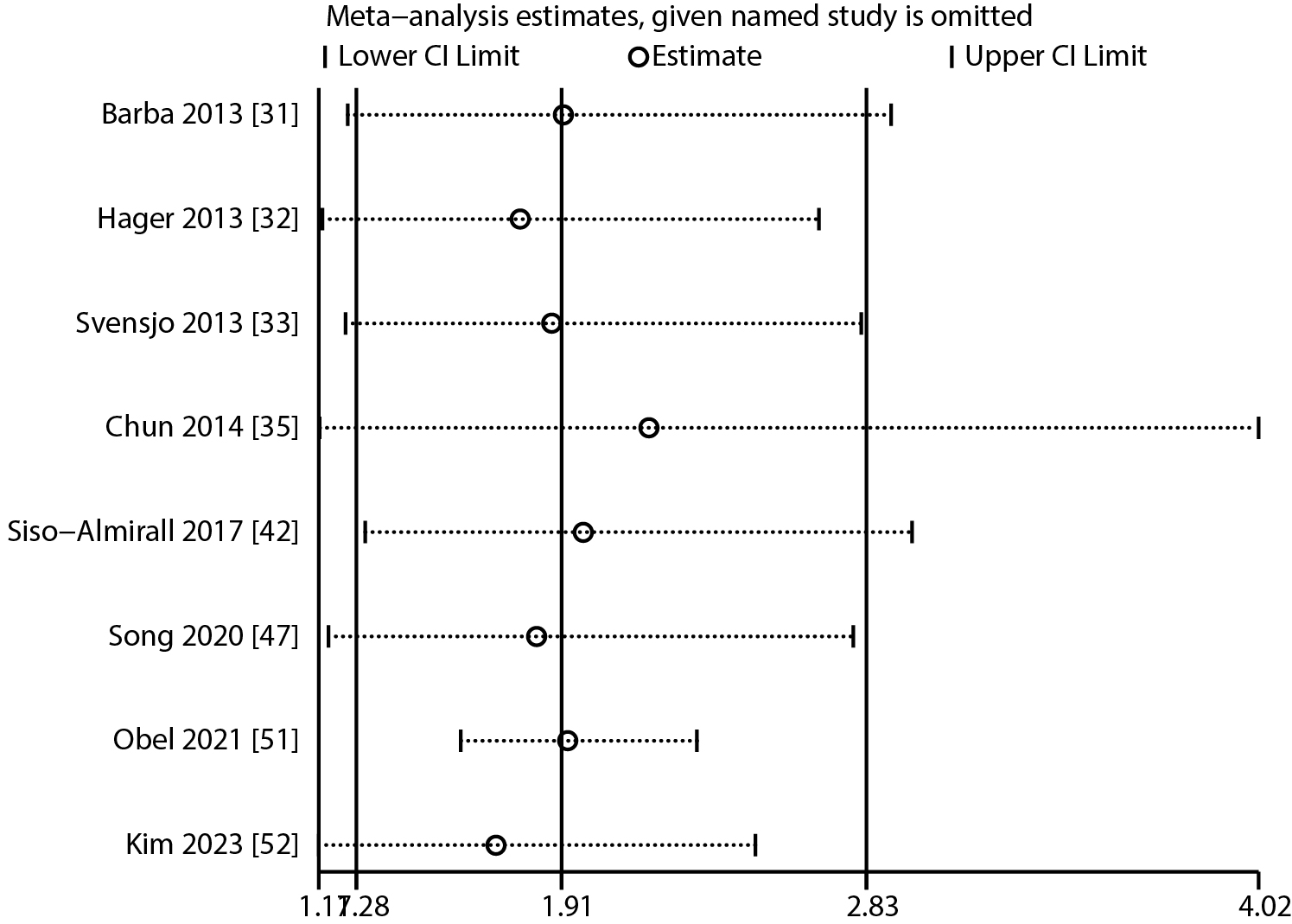

Supplement: S19 Fig — (JPG) [file pone.0329500.s024.jpg]

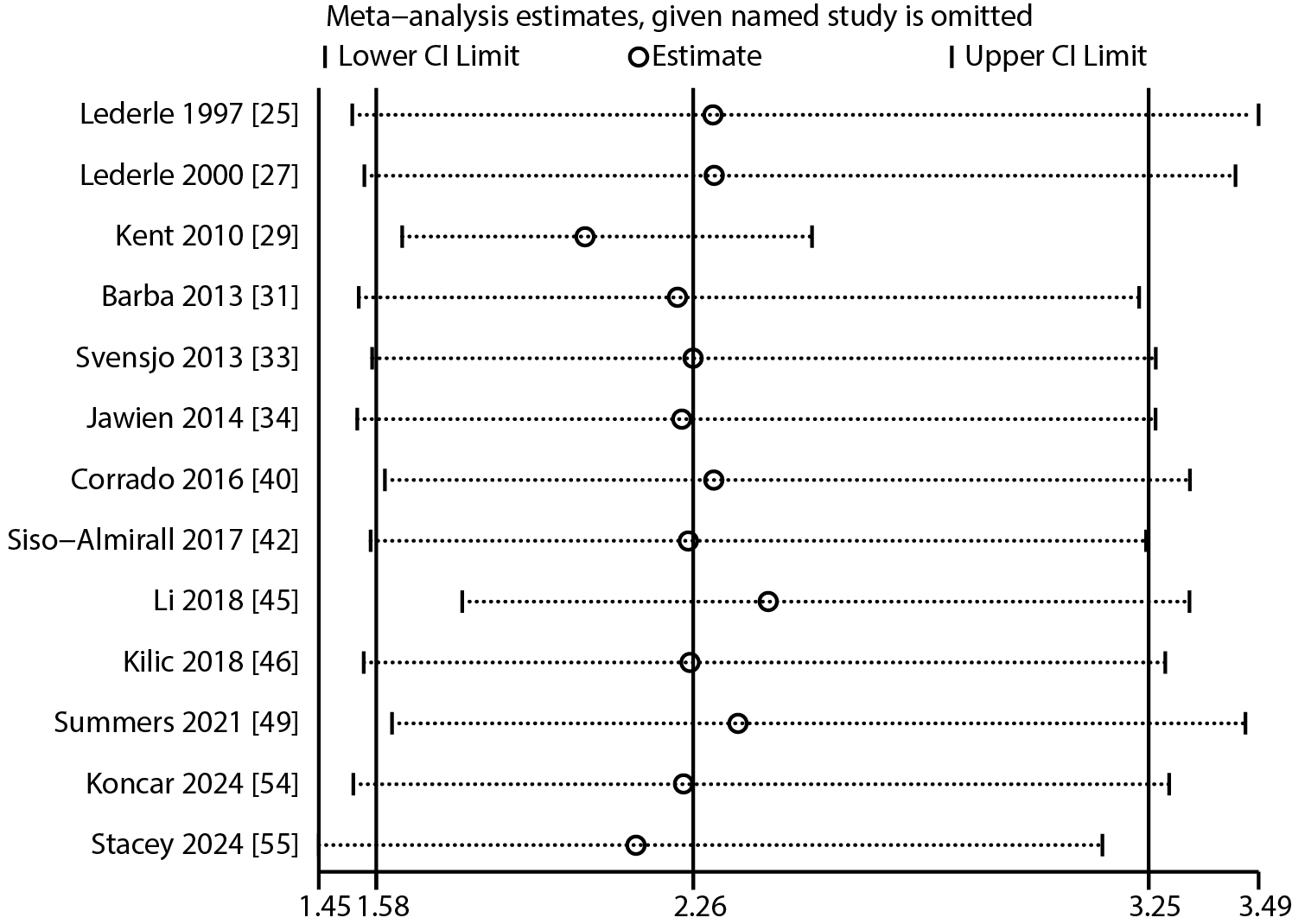

Supplement: S20 Fig — (JPG) [file pone.0329500.s025.jpg]

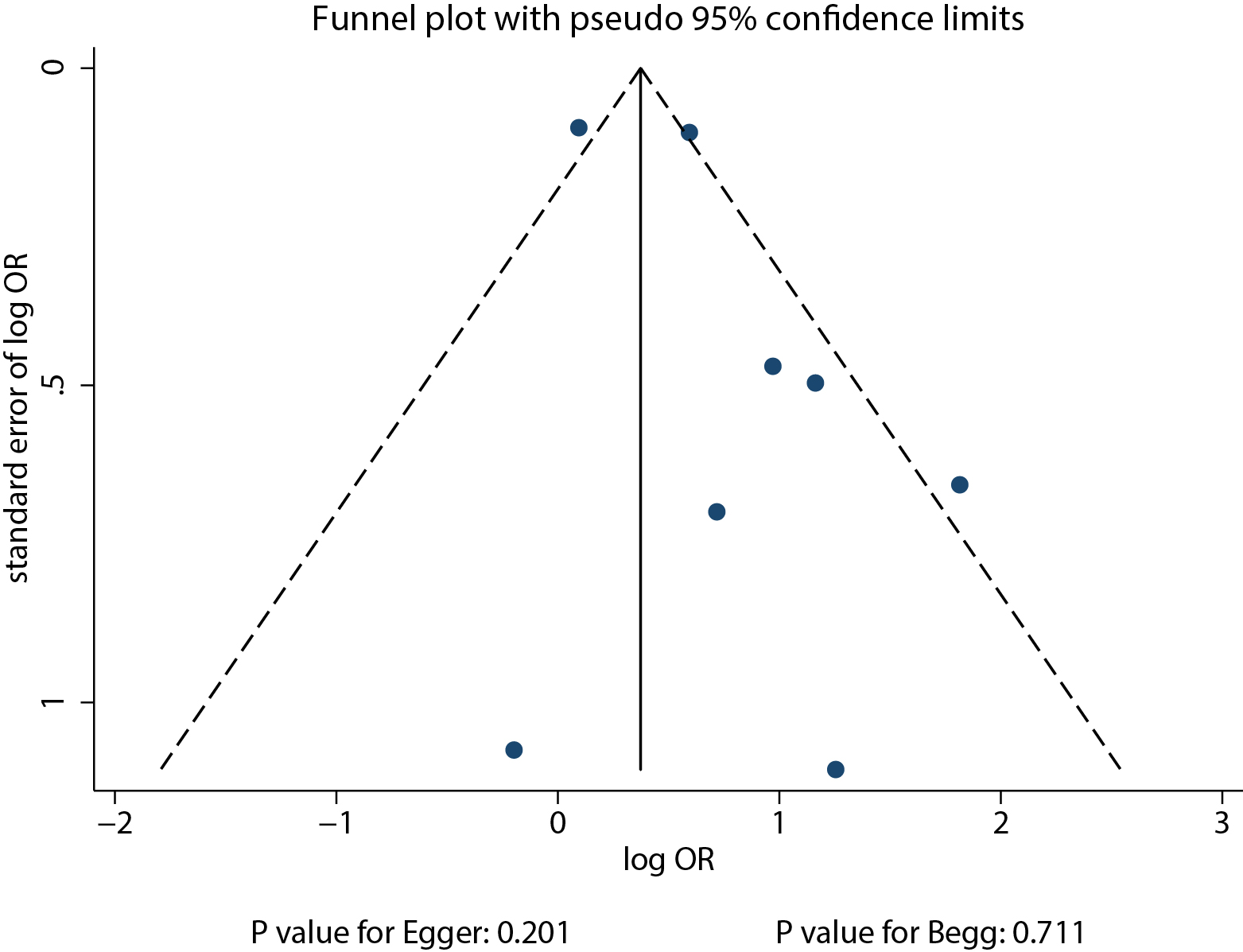

Supplement: S21 Fig — (JPG) [file pone.0329500.s026.jpg]

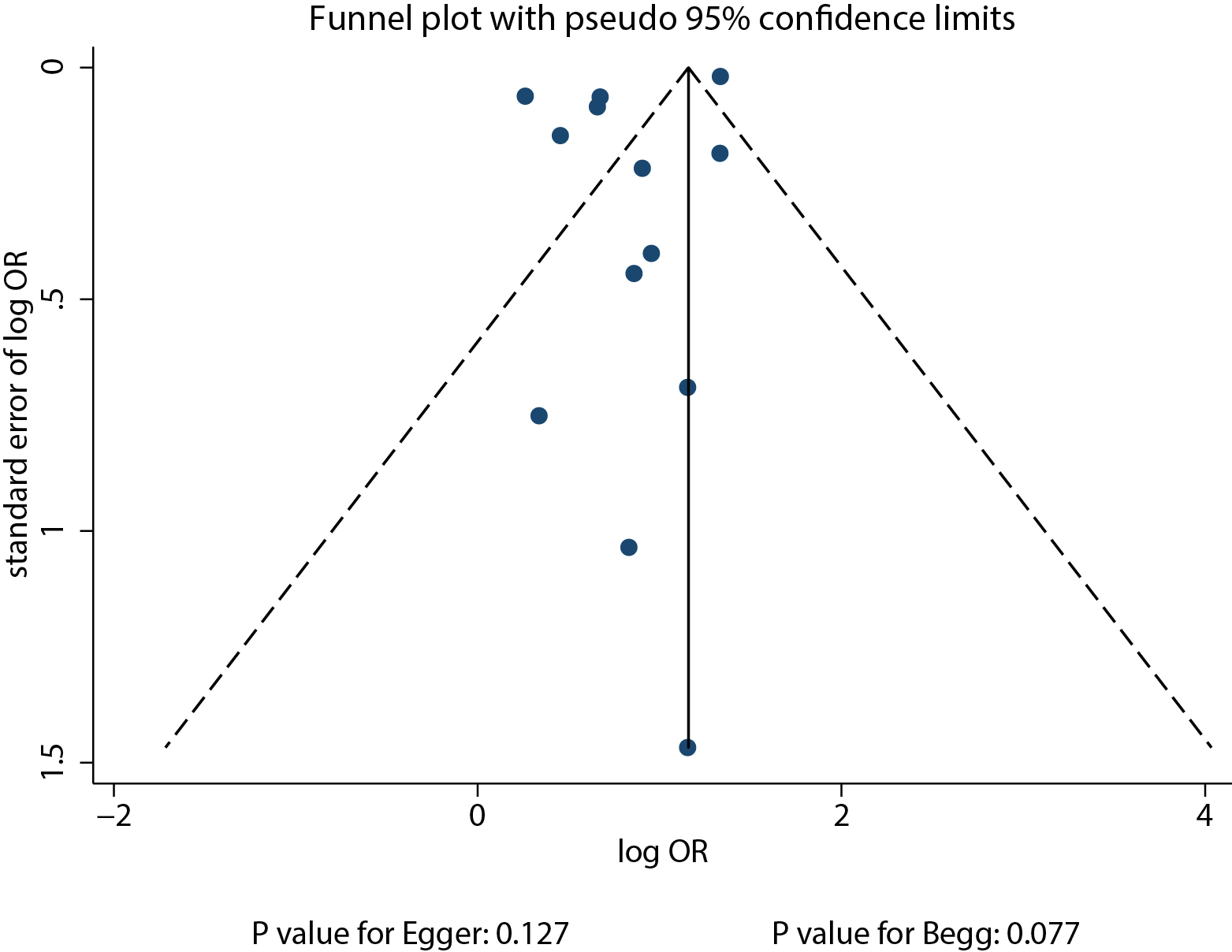

Supplement: S22 Fig — (JPG) [file pone.0329500.s027.jpg]
