# Supplementary material for: Risk factors for abdominal aortic aneurysm in general populations: A systematic review and meta-analysis
Source: PLoS One. 2025 Sep 22;20(9):e0329500. doi: 10.1371/journal.pone.0329500 (PMC12453230; doi:10.1371/journal.pone.0329500)
Supplement: Attachment — Submitted filename: Response to reviewer.docx [file pone.0329500.s029.docx]

PLOS ONE

RE: Manuscript “Risk factors for abdominal aortic aneurysm in general populations: A systematic review and meta-analysis”

Dear editor:

“Risk factors for abdominal aortic aneurysm in general populations: A systematic review and meta-analysis”. We would like to thank *PLOS ONE* for giving us the further opportunity to revise manuscript. We have carefully taken the comments into consideration in preparing our revision, which has resulted in a paper that is clearer and more compelling. The point-by-point responses are attached after this letter. The revisions were highlighted to the text, have been prepared.

The manuscript has not been published previously, in any language, in whole or in part, and is not currently under consideration elsewhere. None of the authors have any competing financial interest to report.

Thank you for considering our manuscript for publication in your esteemed journal.

**Point-By-Point Response**

**Reviewer #1**

***Introduction:***
Question 1: Contextualization of Previous Studies: The introduction provides a general overview of AAA and its risk factors. However, the manuscript would benefit from a more detailed discussion of the gaps in previous studies and the unique contributions of the current study. Please elaborate on the limitations of prior research and explicitly state how this study addresses these gaps.

Response: Thank you for your valuable comments on our manuscript. We fully agree with your suggestion that a more detailed discussion of the limitations of previous studies and the unique contributions of our current study would enhance the quality of the paper. Therefore, we have added this section to our manuscript. ***Please refer to Para 3, section Introduction, as follows:*** “Previous studies focused primarily on a few known risk factors and failed to comprehensively cover all potential risk factors. Although Lampsas et al. [11] reported that the relationship between high lipoprotein levels and increased AAA risk is not affected by racial differences, the current study further explored whether there are significant differences in risk factors among different populations, thereby providing a basis for prevention strategies in various regions. The most recent studies that met the inclusion criteria were included in the analysis”

***Methods:***

Question 1: Search Strategy: While the search strategy is described, the manuscript should include the exact search strings used for each database. This transparency allows for reproducibility of the search process.
Response: Thank you for your valuable feedback. We appreciate your suggestion to enhance the transparency and reproducibility of our search strategy. To address this, we have included the exact search strings used for each database in the manuscript (Supplemental file 1). Here are the details:

**Pubmed:**

(("Abdominal Aortic Aneurysms"[Mesh]) OR "Aneurysms, Abdominal Aortic"[Mesh] OR "Aortic Aneurysms, Abdominal"[Mesh] OR "Abdominal Aortic Aneurysm"[Title/Abstract] OR "Aneurysm, Abdominal Aortic"[Title/Abstract]) AND ("Screening"[Mesh] OR "Mass Screenings"[Mesh] OR "Screening, Mass"[Title/Abstract] OR "Screenings, Mass"[Title/Abstract] OR "Screenings"[Title/Abstract]) AND ("Factor, Risk"[Mesh] OR "Factors, Risk"[Title/Abstract] OR "Risk Factor"[Title/Abstract] OR "Population at Risk"[Title/Abstract] OR "Risk, Population at"[Title/Abstract] OR "Populations at Risk"[Title/Abstract] OR "Risk, Populations at"[Title/Abstract]) NOT "surgical repair"[Title/Abstract]

**EmBase:**

('abdominal aortic aneurysm'/exp OR 'aneurysms, abdominal aortic'/exp OR 'aortic aneurysms, abdominal'/exp OR 'abdominal aortic aneurysm':ti,ab OR 'aneurysm, abdominal aortic':ti,ab) AND ('screening'/exp OR 'mass screenings'/exp OR 'screening, mass':ti,ab OR 'screenings, mass':ti,ab OR 'screenings':ti,ab) AND ('risk factor'/exp OR 'factors, risk':ti,ab OR 'risk factor':ti,ab OR 'population at risk':ti,ab OR 'risk, population at':ti,ab OR 'populations at risk':ti,ab OR 'risk, populations at':ti,ab) NOT 'surgical repair':ti,ab

**Cochrane library:**

(ME "Abdominal Aortic Aneurysms" OR ME "Aneurysms, Abdominal Aortic" OR ME "Aortic Aneurysms, Abdominal" OR "Abdominal Aortic Aneurysm" OR "Aneurysm, Abdominal Aortic") AND (ME "Screening" OR ME "Mass Screenings" OR "Screening, Mass" OR "Screenings, Mass" OR "Screenings") AND (ME "Risk Factors" OR "Factor, Risk" OR "Factors, Risk" OR "Risk Factor" OR "Population at Risk" OR "Risk, Population at" OR "Populations at Risk" OR "Risk, Populations at")

NOT "surgical repair"

Question 2: Study Selection Criteria: The inclusion and exclusion criteria should be more detailed. For example, clarify whether studies without full-text availability were included or excluded, and explain how studies with overlapping populations were handled beyond selecting the most comprehensive one.

Response: Thank you for your valuable feedback. We appreciate your suggestion to provide more detailed inclusion and exclusion criteria. To address your concerns, we have expanded our description of the study selection criteria and provided additional details on how studies with overlapping populations were handled. ***Please refer to Para 2, section Methods, as follows:*** “When multiple studies of the same population were published, the most comprehensive study was selected based on several criteria. First, we compared the studies to confirm whether they involved the same or overlapping populations by examining study periods, locations, and participant characteristics. Next, we evaluated the comprehensiveness of each study by considering factors such as robustness of the study design, sample size, duration of follow-up, and completeness and quality of the data. The study with the largest sample size, longest follow-up period, and the most rigorous methodology was selected to ensure the inclusion of the most reliable and extensive data. Reviews and case report articles were removed because of insufficient data for quantitative analysis. Studies that primarily focused on surgical repair techniques without addressing risk factors or screening were excluded”.
***Results:***

Question 1: Study Characteristics: Provide a more comprehensive table summarizing the characteristics of the included studies (e.g., Outcome investigated, study duration key findings etc.).
Response: Thank you for your valuable feedback. We greatly appreciate your suggestions and have provided a more comprehensive summary of the characteristics of the included studies. To better present this information, we have updated Table 1 to include the study period and the outcomes investigated. Thank you again for your constructive feedback. If you have any further suggestions or need additional information, please let us know.

Question 2: Subgroup Analyses: The manuscript mentions differences in risk factors between Eastern and Western countries but does not provide detailed subgroup analysis results. Include detailed subgroup analysis results and discuss any significant variations found.

Response: Thank you for your valuable feedback. We appreciate your suggestion to include detailed subgroup analysis results and discuss any significant variations found, particularly regarding the differences in risk factors between Eastern and Western countries. While our primary aim was to explore a broad range of risk factors across all included studies, we understand the importance of providing a more detailed subgroup analysis to address the specific differences you mentioned. However, we did not perform a more detailed subgroup analysis in the initial manuscript because our research direction was focused on a comprehensive exploration of all risk factors rather than on a single factor.
***Discussion:***
Question 1: Implications for Practice and Policy: The manuscript concludes that early intervention and health education are necessary but lacks specific recommendations. Provide more detailed suggestions for clinical practice and public health policies based on the study’s findings.
Response: Thank you for your valuable feedback. We appreciate your suggestion to provide more detailed recommendations for clinical practice and public health policies based on our study's findings. Below, we have included specific suggestions that align with the identified risk factors for AAA. ***Please refer to Para 2, section Discussion, as follows:*** “These findings highlight the importance of early intervention and health education for improving modifiable risk factors to reduce the risk of AAA, primarily including: (1) implementing targeted screening programs for high-risk populations, particularly males over 65 years of age, current or former smokers, and individuals with a family history of AAA; (2) establishing regular follow-up protocols to monitor the progression and provide early intervention if necessary for patients identified with small AAAs; (3) risk factor management, including smoking cessation, and hypertension and dyslipidemia management; and (4) health education regarding lifestyle modifications and campaigns to promote awareness of signs and symptoms of AAA”.

Question 2: Limitations: The limitations section should be expanded. Discuss potential biases in study selection, limitations in the quality of included studies, and the potential impact of these limitations on the findings.

Response: Thank you for your valuable feedback. We appreciate your suggestion to expand the limitations section to discuss potential biases in study selection, limitations in the quality of included studies, and the potential impact of these limitations on the findings. ***Please refer to Para 5, section Discussion, as follows:*** “This study has several limitations that should be considered when interpreting the findings. First, we included a combination of cross-sectional, retrospective, and prospective studies. This heterogeneity in study design may have introduced selection bias, as different study types have varying levels of methodological rigor and are subject to different types of bias. Moreover, retrospective studies, in particular, are susceptible to recall bias, whereby participants may not accurately remember past exposures or events, potentially leading to misclassification of risk factors. Furthermore, the inclusion of observational studies means that uncontrolled confounding factors may have influenced the results. These confounders could not be fully accounted for in the meta-analysis, potentially leading to biased estimates of the association between risk factors and AAA. Second, most of the included studies followed a cross-sectional design, which limited our ability to establish causality. Moreover, the methodological quality of the included studies varied. Some studies had small sample sizes, limited follow-up periods, and inadequate control of confounding variables, which may have affected the reliability and generalizability of the findings. Third, there was substantial heterogeneity in most of the identified risk factors, which could not be fully explained by sensitivity and subgroup analyses. Finally, as with any meta-analysis, there is the potential for publication bias, whereby studies with significant or positive results are more likely to be published. Moreover, the meta-analyses were based on published articles, which restricted detailed analyses. Further large-scale prospective studies are required to verify the findings of this study and address the limitations mentioned above. Prospective studies with standardized data collection methods and rigorous control of confounding factors would help establish causality and provide more reliable estimates of risk factors for AAA”

**Reviewer #2**

General comments: In this manuscript, the authors conducted a systematic review and meta-analysis on AAA, which is a common topic in vascular surgery. However, there are many similar systematic reviews and meta-analysis on AAA have already been published, and the majority of risk factors presented in this manuscript have been examined by other analysis, undermining the importance of the current study.
Response: Thank you for your valuable feedback. We appreciate your concern regarding the novelty and importance of our systematic review and meta-analysis on AAA. We understand that there are several existing systematic reviews and meta-analyses on this topic, and many of the risk factors we examined have been previously studied. However, we believe that our study adds significant value to the existing literature for the following reasons: (1) our study includes a more comprehensive and up-to-date analysis, incorporating recent studies that have not been included in previous reviews. This ensures that our findings reflect the most current evidence available, which is crucial given the ongoing advancements in vascular surgery and research; (2) unlike many previous reviews, our study includes a mix of cross-sectional, retrospective, and prospective studies. This diversity in study designs allows for a more robust and comprehensive understanding of the risk factors associated with AAA, as different study types can provide complementary insights; (3) we conducted detailed subgroup and sensitivity analyses to explore the sources of heterogeneity and to assess the robustness of our findings. These analyses provide a deeper understanding of the variations in the data and help to identify specific subgroups where the risk factors may have a more pronounced effect; and (4) we conducted detailed subgroup and sensitivity analyses to explore the sources of heterogeneity and to assess the robustness of our findings. These analyses provide a deeper understanding of the variations in the data and help to identify specific subgroups where the risk factors may have a more pronounced effect.

Question 1: Novelty. What is the novelty of this current research? Most of these have been reported by other papers.
Response: Thank you for your valuable feedback and for raising the question about the novelty of our current research. We understand your concern that many of the risk factors we have examined have been reported in previous studies. However, we believe that our study makes several important and novel contributions to the existing literature on AAA. Our systematic review and meta-analysis include the most recent studies, some of which have not been included in previous reviews. Moreover, we conducted detailed subgroup and sensitivity analyses to explore the sources of heterogeneity and to assess the robustness of our findings. Furthermore, our study incorporates a mix of cross-sectional, retrospective, and prospective studies. This diversity in study designs allows us to provide a more robust and holistic view of the risk factors, as different types of studies can offer unique insights and complement each other's findings.

Question 2: Because most of these have been reported repeatedly, I suggest the authors focus on the limitations of other meta-analysis while doing their own analysis.
Response: Thank you for your valuable feedback and for suggesting that we focus on the limitations of other meta-analyses while conducting our own. We appreciate your insight and agree that addressing the limitations of previous studies can add significant value to our work. ***Please refer to Para 3, section Introduction, as follows:*** “Previous studies focused primarily on a few known risk factors and failed to comprehensively cover all potential risk factors. Although Lampsas et al. [11] reported that the relationship between high lipoprotein levels and increased AAA risk is not affected by racial differences, the current study further explored whether there are significant differences in risk factors among different populations, thereby providing a basis for prevention strategies in various regions. The most recent studies that met the inclusion criteria were included in the analysis”

Question 3: There are multiple statements in the manuscript seeming to be more arbitrary than fact based. For example, line237-239:"AAA was more prevalent in males than in female, which could explained by the arterial wall in males is thinner and weaker, making it more susceptible to conditions like atherosclerosis, thus increasing the risk of AAA." what is the evidence for the claim that the arterial wall in males is thinner and weaker?
Response: Thank you for your thorough review and for pointing out the need for evidence to support the statement regarding the arterial wall in males. We appreciate your attention to detail and agree that all claims in the manuscript should be supported by robust evidence. We have revised the statement to reflect the current evidence and to avoid making unsupported claims. The revised text now reads: "Guirguis-Blake et al. [3] indicated that AAA is more prevalent in males than in females, which could be explained by several factors, including differences in hormonal profiles, smoking prevalence, and other lifestyle factors [53, 54]. Further research is required to fully understand the underlying mechanisms. "

Question 4: Multiple grammar mistakes need to be corrected.

Response: Thank you for pointing out the grammatical errors in the manuscript. We greatly appreciate your feedback and have carefully proofread and corrected the manuscript. Here are the specific steps we have taken: (1) we have conducted a thorough grammatical and spelling check of the entire manuscript to ensure that all sentences adhere to English grammar rules; and (2) to further enhance the quality of the manuscript, we have had it reviewed and edited by a professional English editor from Editage. All changes made by the editor are shown in the revised document using track changes. We hope these revisions address the grammatical issues and improve the overall quality of the manuscript. Thank you again for your valuable feedback. If you have any further suggestions or need additional information, please let us know.
